# Supplementary material for: Treatment Strategies Guided by [18F]FDG-PET/CT in Patients with Locally Advanced Cervical Cancer and [18F]FDG-Positive Lymph Nodes
Source: Cancers (Basel). 2024 Feb 8;16(4):717. doi: 10.3390/cancers16040717 (PMC10887300; doi:10.3390/cancers16040717)
Supplement: Supplementary file 1 [file cancers-16-00717-s001.zip › cancers-2733740-supplementary.pdf]

Table S1. Baseline characteristics according to nodal treatment by nodal boosting, extended-field radiotherapy, and debulking separately.

	Boosting						Extended-field radiotherapy						Debulking					
**Baseline characteristics**	**M**	**Without (n=62)**		**With (n=320)**		**p-value**	**M**	**Without (n=24)**		**With (n=86)**		**p-value**	**M**	**Without (n=382)**		**With (n=52)**		**p-value**
Median age, years	0	50	(22-88)	49	(23-82)	0.33	0	47	(27-82)	51	(22-79)	0102	0	49	(22-88)	50	(25-77)	0.26
Median body mass index, kg/m^2	21	25	(15-36)	24	(15-77)	0.51	4	23	(19-77)	25	(16-44)	0.51	23	24	(15-77)	26	(17-39)	0.33
Charlson Comorbidity Index	58						17						70					
0		40	78.8%	216	78.8%	1.00		18	85.7%	55	76.4%	0.53		256	79.0%	33	82.5%	1.00
1		8	16.8%	46	16.8%			3	14.3%	10	13.9%			54	16.7%	6	15.0%	
≥2		2	4.0%	12	4.4%			0	0.0%	7	9.7%			14	4.3%	1	2.5%	
FIGO 2009 stage	0						0						0					
IB2		3	4.8%	46	14.4%	0.11		2	8.3%	8	9.3%	0.23		49	12.8%	10	19.2%	0.65
IIA2		1	1.6%	13	4.1%			2	8.3%	4	4.7%			14	3.7%	3	5.8%	
IIB		31	50.0%	169	52.8%			14	58.3%	39	45.4%			200	52.4%	24	46.2%	
IIIA		3	4.8%	11	3.4%			2	8.3%	2	2.3%			14	3.7%	1	1.9%	
IIIB		18	29.0%	61	19.1%			2	8.3%	23	26.7%			79	20.7%	12	23.1%	
IVA		6	9.7%	20	6.3%			2	8.3%	10	11.6%			26	6.8%	2	3.9%	
Median tumour size, mm	20	56	(24-220)	50	(20-105)	0.10	10	50	(25-85)	50	(30-105)	0.97	21	50	(20-220)	55	(38-100)	0.017*
Histological subtype	0						0						0					
Squamous cell carcinoma		54	87.1%	280	87.5%	0.93		18	75.0%	75	87.2%	0.11		334	87.4%	48	92.3%	0.65
Adeno(squamous) carcinoma		7	11.3%	33	10.3%			3	12.5%	9	10.5%			40	10.5%	4	7.7%	
Other carcinomas		1	1.6%	7	2.2%			3	12.5%	2	2.3%			8	2.1%	0	0.0%	
Additional imaging techniques	0						0						0					
CT		16	26.0%	82	25.6%	1.00		7	29.2%	34	39.5%	0.48		98	25.7%	22	42.3%	0.020*
MRI		57	91.9%	304	95.0%	0.36		23	95.8%	77	89.5%	0.69		361	94.5%	44	84.6%	0.014*
Status of [^{18}F]FDG-positive node	0						0						0					
Suspicious		54	87.1%	302	94.4%	0.051		22	91.7%	79	91.9%	1.00		356	93.2%	52	100.0%	0.058
Inconclusive		8	12.9%	18	5.6%			2	8.3%	7	8.1%			26	6.8%	0	0.0%	

FDG-positive nodes per region [1]																		
Pelvic	1	61	98.4%	314	98.4%	1.00	1	21	87.5%	82	96.5%	0.12	1	375	98.2%	51	98.1%	0.59
Common iliac	6	6	9.7%	53	16.9%	0.18	3	21	87.5%	38	45.8%	<0.001*	6	59	15.7%	14	26.9%	0.050
Para-aortic	6	7	11.5%	60	19.1%	0.20	1	4	16.7%	63	74.1%	<0.001*	6	69	17.8%	19	36.5%	0.003*
Median short-axis of suspicious node, mm [2]	83	10	(6-26)	12	(6-86)	0.02*	48	8	(6-16)	10	(4-33)	0.31	85	12	(6-86)	21	(9-50)	<0.001*

[1] patients may have positive lymph nodes in multiple regions,

[2] concerns common iliac and para-aortic nodes for extended-field radiotherapy analysis,

* statistically significant.

Abbreviations: M, missing; FIGO, International Federation of Gynaecology and Obstetrics; CT, computed tomography; MRI, magnetic resonance imaging; FDG, fluoro-D-glucose.
